# How do adolescents classify foods as healthy and unhealthy?: A qualitative inquiry from rural India

**DOI:** 10.1017/jns.2023.101

**Published:** 2023-11-20

**Authors:** Sangeeta Kansal, Aryan Raj, Kumari Smita, Anthony Worsley, Neha Rathi

**Affiliations:** 1 Department of Community Medicine, Institute of Medical Sciences, Banaras Hindu University, Varanasi 221005, Uttar Pradesh, India; 2 Department of Psychiatry, Government Medical College and Hospital, Chandigarh 160030, India; 3 School of Exercise and Nutrition Sciences, Deakin University, Geelong, VIC 3220, Australia; 4 Department of Home Science, Mahila Mahavidyalaya, Banaras Hindu University, Varanasi 221005, Uttar Pradesh, India

**Keywords:** Adolescents, Food, Food choice, Health eating, India, Qualitative research methods

## Abstract

Indian adolescents exhibit obesogenic dietary habits including low intake of fruits and vegetables and increasing consumption of fast food and carbonated beverages. Adolescents’ classification of healthy and unhealthy foods is likely to have significant implications for their dietary behaviour, and consequently, their health. However, there is limited evidence about the ways Indian adolescents classify foods as healthy or unhealthy. Hence, this qualitative study was designed to explore how Indian adolescents classify nutritious or non-nutritious food choices. Convenience sampling was used to recruit the study participants from Tikari village in Uttar Pradesh, India. Underpinned by the social constructivist lens, the adolescents were interviewed face-to-face in Hindi. All the interactions were digitally recorded, transcribed verbatim, and translated into English prior to data analysis. Transcribed data were analysed thematically using inductive as well as deductive coding, and subsequently, conceptual themes were extracted. A total of thirty-nine adolescents (twenty boys; nineteen girls) aged 10–19 years participated in this qualitative inquiry. The adolescents classified healthy and unhealthy foods based on the following six themes: (i) Food groups and nutrients; (ii) Health and immunity; (iii) Type of ingredient; (iv) Place and time of food preparation; (v) Packaging; and (vi) Parental influence. These findings can inform school-based food literacy interventions to foster healthy dietary habits and cooking skills among Indian adolescents.

## Introduction

Adolescence is regarded as a critical transition phase in the lifespan^(1)^ with implications for adult health and economic productivity of nations. Spanning from approximately 10–19 years of age, adolescence is characterised by dramatic physiological and psychological changes^(1)^ with augmented nutritional demands.^(2)^ Culinary transition coupled with globalisation and economic growth has resulted in the development of unhealthy dietary habits among adolescents worldwide.^(2–4)^ Indeed, Indian adolescents exhibit poor dietary intakes (i.e. increased intakes of high-energy nutrient poor foods and sugar-sweetened beverages)^(5–7)^ and unhealthy food practices (e.g. skipping breakfast).^(7,8)^ These unhealthy dietary patterns inculcated in adolescence often track into adulthood.^(2)^ Moreover, this overreliance on Westernised diets and reduced intake of home cooked food has adverse health consequences including obesity^(6)^ and diet-related chronic degenerative diseases.^(9–12)^

Adolescent overweight and obesity are one of the most severe public health crises of the twenty-first century^(13)^ with a burgeoning prevalence in low-middle-income countries including India.^(14–17)^ Adolescent obesity is largely prevalent in the metropolitan areas^(18,19)^; however, over the last few years, an escalation in obesity prevalence has also been noted in rural settings.^(20,21)^ This rise is evident in the findings of a nationwide survey which showed that overweight or obesity (BMI ≥ 25⋅0 kg/m^2^) increased from 14⋅3 % (2015–16) to 19⋅3 % (2019–21) in rural men (aged 15–49 years) and from 15 % (2015–16) to 19⋅7 % in rural women (aged 15–49 years).^(22)^ Likewise, an upsurge has been reported in the blood pressure and blood sugar levels among the rural population.^(22)^ Findings from the longitudinal tracking of adolescent food choice behaviours^(23)^ and obesity into adulthood,^(24)^ suggest that it is important to adopt healthy eating strategies during the critical pubertal phase.^(25)^

One such healthy eating strategy is to motivate young people to make informed food choices. Nutrition knowledge is often viewed as a vital predisposing factor for the adoption of healthy eating habits.^(26)^ In fact, several behavioural interventions and public health programmes have been successful in improving food choices through boosting nutrition knowledge.^(27,28)^ Besides influencing perceptions about healthy eating, nutrition knowledge also serves as a key element in determining individuals’ food classification criteria and subsequently their food choices.^(29,30)^ Therefore, to improve Indian adolescents’ nutritional status and alleviate their chronic disease risk, it is imperative to explore whether adolescents are aware of healthy and unhealthy food items and further investigate the food classification criteria they employ while selecting foods.

Although empirical evidence about young peoples’ perceptions of food classification and food choices is expanding,^(31–33)^ very limited evidence exists in relation to Indian adolescents^(34)^ and more so in the rural context. This lack of research presents an opportunity to understand Indian adolescents’ food classification criteria to inform future public health interventions to promote healthy eating practices among them. Therefore, this qualitative study aimed to assess the ways adolescents in rural India classify foods as healthy and unhealthy.

## Methods

This study was conducted according to the guidelines laid down in the Declaration of Helsinki and all procedures involving human subjects were approved by the Institutional Ethical Committee at the Institute of Medical Sciences, Banaras Hindu University (Dean/2021/EC/2817). Written informed consent was obtained from all subjects. All aspects of the research protocol have been reported (see Supplementary file) in accordance with the Consolidated Criteria for Reporting Qualitative Research (COREQ) – a 32-item checklist.^(35)^ A qualitative research approach guided by a social constructivism framework was employed in this study. This epistemological framework allowed the study investigators to anticipate and appreciate the respondents’ perspectives about the topic which resulted from their interactions with the members of the community.^(36)^ Based on this interpretive paradigm, the authors explored the adolescents’ experiences regarding healthy and unhealthy foods consumed by them on a daily basis.

Within the context of the present study, NR is an early-career researcher specialising in behavioural nutrition and qualitative research methods, conducted all interviews. She did not have any previous relationship with the study respondents. Other members of the research team included two medical doctors (KS, SK) with a background in public health and family medicine, one university student (AR) pursuing his post graduate studies in social work and a food psychologist (AW). AR was engaged in taking field notes during the interview sessions and he also transcribed all the audio records to ensure consistency. The transcripts were translated to English by KS and she further assisted NR in data analysis. Both SK and AW being senior researchers, supervised the analysis, interpretation and reporting of the themes. The involvement of researchers from various academic fields diminished the possibility of any individual or disciplinary biases during the interpretation of the data.

Adolescents aged 10–19 years formed the study sample because food habits developed in the pubertal phase often track into adulthood^(23)^ and therefore it becomes imperative to understand how adolescents practice food selection. Moreover, Indian adolescents aged 10–19 years are prone to overweight and obesity^(22)^ which is triggered by excessive consumption of energy dense, nutrient-poor foods, and sugar-sweetened beverages.^(5–7)^ In addition, adolescents aged 10–19 years serve as direct beneficiaries of nearly all the government health programmes targeting adolescents,^(37)^ furthering endorsing the selection of adolescents. Adolescents aged 10–19 years residing in Tikari village were eligible to participate in this qualitative inquiry. The study participants were recruited from Tikari village (Tikari is situated in Varanasi district in the state of Uttar Pradesh; Uttar Pradesh is the most populous state in India) using convenience sampling. As per 2011 Census data, Tikari had a population of 5431, out of which 2830 were males and 2601 were females.^(38)^ A total of 782 families were residing in the village.^(38)^ There were 411 male children and 389 female children between the age 0–6 years.^(38)^ The literacy rate was 73⋅31 %. Agriculture was recognised as the primary occupation of the villagers.^(38)^ A social worker employed in the Department of Community Medicine of the Institute of Medical Sciences, Banaras Hindu University, where the current study was conceptualised and conducted, assisted in identification of households with adolescents. Consequently, the lead researcher (NR) met the participants and their parents in person to explain to them about the research protocol as well as sought their approval for participation in the inquiry.

Two open-ended questions (Table 1) were derived from the literature^(39)^ and were translated to Hindi by NR (principal investigator fluent in both Hindi and English) and they were further checked by another author (SK). The translated questions were pre-tested with two male and two female adolescents, and no revision was needed. The data from these four preliminary interviews were merged with the final data set.

**Table 1.** Interview questions

Q1	Which among the meals that you consume are the healthiest and the unhealthiest and why? Probe: On what basis do you classify meals as healthy or unhealthy? Can you give examples.
Q2	Which among the snacks that you consume are the healthiest and the unhealthiest and why? Probe: On what basis do you classify snacks as healthy or unhealthy? Can you give examples.

Face-to-face interviews were conducted in the Hindi language from November 2022 to January 2023 by NR in the presence of a note keeper (AR). Interviews were carried out in a quiet and open area in the village where no parent or peer known to the adolescent were present. The interactions were recorded using an audio recorder with the consent of the interviewees. The interview duration ranged from 13 to 21 min. At the 36th interview, data saturation^(40,41)^ was achieved. Data saturation is the point when no new additional thematic information is obtained after a number of interviews have been carried out.^(40)^ Nevertheless, the three remaining scheduled interviews were carried out, and the data collection was concluded at the 39th interview. All the interviewees received fresh seasonal fruits (e.g. guava, oranges, banana) and plain yoghurt for their participation.

Thematic analysis was conducted concurrently with data collection to maintain a balance between the two processes.^(42)^ Interview recordings were transcribed verbatim by the notekeeper (AR) and translated to English by another author (KS) who is fluent in both Hindi and English. The interviewer (NR) checked a sample of fifteen transcripts for accuracy. The transcripts were uploaded to the NVivo (Version 12) software program (QSR International Pvt Ltd. 2018) and analysed utilising the Template Analysis Technique. In this approach, transcribed data is analysed using the following six steps: (i) Repeated reading of the transcripts to gain familiarity with the data; (ii) Conduct of initial coding of the data, i.e. identification of some ‘*a priori*’ themes based on the research question and review of the literature; (iii) Hierarchical organisation of themes (narrower themes are nested within broader ones) between the emerging themes and organising them into meaningful clusters; (iv) Design of an initial version of the template (i.e. a set of codes); (v) Application of the initial coding template and revision if required; and (vi) Creation of a final version of the template and application of it to the rest of the data.^(43)^ NR analysed all the transcripts while the second coder (KS) reviewed 50 % of the data set to improve reliability and minimise any personal bias associated with interpretation of the data.^(44)^ In case of the difference of opinion, the template was modified through mutual discussion.^(45)^ Inter-rater reliability was verified by two professionals, i.e. one Home Economist and one health psychologist who independently analysed five transcripts each.^(46)^ Both data analysis and interpretation were underpinned by a social constructivism paradigm as the analysis was based on the experiences shared by the participants, and subsequently, these experiences are shared verbatim as quotes to support the emerging themes. A comprehensive analysis of the transcribed data representing the template themes and descriptive quotations from the interviewees is provided in the Results section.

## Results

Thirty-nine adolescents aged 10–19 years were interviewed. Out of these thirty-nine adolescents, twenty were boys. With the exception of one student (A1, an early school leaver), all the other adolescents were studying between 6th and 12th grades. Thirty-seven adolescents attended public school, while one female adolescent (A2) attended an independent school. The mean age of the participants was 14⋅35 years (sd = 2⋅70).

Thematic analysis revealed that the adolescents classified healthy and unhealthy foods based on the following six themes: (i) Food groups and nutrients; (ii) Health and immunity; (iii) Type of ingredient; (iv) Place and time of food preparation; (v) Packaging; and (vi) Parental influence. These themes along with relevant quotes are described below:

### Theme 1: Food groups and nutrients

All the interviewees perceived a balanced diet to be healthy as it comprised all the food groups like cereals, fruits, vegetables, pulses and legumes, milk, eggs, and nuts. They reported that these food groups are vital for the functioning of the human body as they provide essential nutrients like protein, vitamins, and minerals. Besides providing nutrients, a balanced diet also satisfies hunger as suggested by our interviewees. Fast foods like pizza, burger, and deep-fried Indian snacks were not considered healthy since they were devoid of nutrients, as described by the adolescents. These views are expressed through the quotations below:

‘*The food that we eat is a balanced meal and it keeps our body healthy. We get protein and vitamins from it.*’ (A39, 15 years, F, 9th Grade)

‘*The body remains healthy by taking a balanced diet …  I drink milk everyday. All types of vitamins are found in milk. It provides protein. It is beneficial to eat green vegetables. Packaged food is harmful, it does not provide energy … .*’ (A23, 18 years, M, 12th Grade)

‘*Lentils is good for our health which gives us strength and energy …  and by consuming green vegetables our body will remain healthy*.’ (A14, 17 years, F, 11th Grade)

‘*I eat vegetables and chapatis (Indian bread) daily …  It provides nutrition. It gives strength to our body. Hunger gets satisfied …* ’ (A19, 12 years, M, 7th Grade)

‘*Cashew, almonds and raisins are nutritious. It contains protein. Lentils, rice, chapatis, and vegetables fill the stomach. It will give me energy. Eating Punjabi Tadka (deep fried Indian snack made from potatoes) does not provide any strength … .*’ (A27, 13 years, M, 7th Grade)

‘*Green vegetables, milk, eggs, and lentils are nutritious …  Green vegetables provide iron.’* (A37, 19 years, F, 12th Grade)

### Theme 2: Health and immunity

In conjunction with Theme 1, the adolescents often discussed Theme 2, i.e. health and immunity. The participants claimed that foods like roti (Indian bread), dal (pulses), vegetables, fruits, and nuts provide immunity to the body and keep the body free of ailments while consumption of burgers, pizza, chips, deep-fried Indian snacks is detrimental to health. It can result in jaundice and liver damage since these foods contain excess oil and spices, as reported by the participants.

‘*The things that are available in the market like pizza and burger harms our body*.’ (A8, 14 years, M, 7th Grade)

‘*Kurkure, mixture (packaged deep fried Indian snack), & biscuits are not nutritious as it damages the liver*.’ (A22, 18 years, M, 11th Grade)

‘*If we eat noodles, it can harm us because it is made of refined flour. There may be several diseases, the liver may get damaged*.’ (A29, 15 years, M, 8th Grade)

‘*Eating samosa, Kurkure, namkeen (deep fried Indian snack) can cause many types of diseases or illness in our body like jaundice can happen*.’ (A14, 17 years, F, 11th Grade)

‘*Amla (Indian Gooseberry) provides Vitamin C. It will not cause any disease*.’ (A35, 15 years, F, 10th Grade)

‘*Fruits and green vegetables are healthy as they give us ability to fight against diseases while fast food is unhealthy* … .’ (A26, 11 years, M, 7th Grade)

### Theme 3: Type of ingredient

The type of ingredients used in food preparation were cited as important factors when choosing between healthy and unhealthy foods. Food prepared at home was considered nutritious because it was prepared using fresh produce cultivated in their fields while food prepared in restaurants or packaged food were not regarded as healthy as they could be adulterated with toxic and inferior quality substances including chemicals.

‘*Sometimes I eat chips, biscuits, Kurkure. It is not nutritious as we are not aware what it is made of and what ingredients are used*.’ (A2, 15 years, F, 8th Grade)

‘*Outside food like chips is always cooked in oil and it is not known what it is made of; if we eat it daily, we can get sick* … .’ (A19, 12 years, M, 7th Grade)

‘*Packaged food can be adulterated which can affect our body, it can cause illness* … .’ (A38, 13 years, F, 9th Grade)

‘*There is plastic inside chips and Kurkure, it is made of chemicals* … ’ (A30, 15 years, M, 8th Grade)

‘*Lentils and rice are good because it grows in our own field, it is cultivated by us, it is fresh* … .’ (A6, 14 years, F, 8th Grade)

### Theme 4: Place and time of food preparation

Unanimously, the participants mentioned that anything prepared at home was healthy while anything prepared outside, for example, the market was unhealthy. They further reported that homemade food was fresh and hygienic. The interviewees also noted that time of preparation was critical in determining whether a particular meal or snack was healthy or unhealthy. They reported that they were not sure when the packaged food was packed and therefore were hesitant in purchasing or consuming it.

‘*The pulses and vegetables that we cook at home are harvested from the field and we use it fresh, so it is good!*’ (A32, 19 years, M, 12th Grade)

‘*I do not eat packaged food because I do not know how many days before it is made so one should eat home cooked food as it is prepared with hygiene* … .’ (A1, 16 years, F, Early school leaver)

‘*I eat chips once in two weeks. I eat very little because it is cooked since many days and the date has also expired* … .’ (A21, 17 years, M, 10th Grade)

‘*Sometimes I eat chips but we do not know how and when it is made* … ’ (A24, 14 years, F, 7th Grade)

‘*Lentils and rice are nutritious because it is home food and grown in the field*.’ (A8, 14 years, M, 7th Grade)

‘*We should eat homemade food and not outside food. Homemade food is cooked with hygiene and so it is good for our health*.’ (A14, 17 years, F, 11th Grade)

### Theme 5: Packaging

The type of packaging material was used by the participants to classify whether a food item was healthy or unhealthy. Food items packed in plastic were not considered healthy as illustrated by the following two quotes:

‘*Outside food is packed in plastic so it is not healthy!*’ (A39, 15 years, F, 9th Grade)

‘*Chips does not provide any nutrition because it remains inside plastic*.’ (A6, 14 years, F, 8th Grade)

### Theme 6: Parental influence

A few interviewees classified food as healthy or unhealthy based on their parents’ recommendations. They noted that their parents did not purchase foods like chips, cakes, fried Indian snacks such as samosa and also prohibited them (i.e. the adolescents) from either purchasing or consuming these foods.

‘*Papa says that we should not eat outside food as it is not good for our body*….’ (A10, 10 years, F, 6th Grade)

‘*I am not allowed to eat anything from the market. Papa has forbidden me from purchasing and eating*.’ (A17, 11 years, M, 7th Grade)

‘*Sometimes when I feel like eating chips, I buy them. However, not much, very little as everyone refuses at home. They say that it will cause illness*.’ (A13, 17 years, F, 11th Grade)

## Discussion

This study provides first-hand information on how Indian adolescents classify nutritious and non-nutritious foods. The key findings were that adolescents used food groups and nutrients along with immunity to classify healthy and unhealthy foods; nutritious and non-nutritious foods were also classified on the basis of type of ingredients, packaging material as well as place and time of food preparation; and finally, parental influence was used as a classification factor.

Foods were categorised as nutritious or non-nutritious depending on the nutrient composition and food groups as reported by the interviewees. They further noted that food items containing vital nutrients like proteins, vitamins, and minerals were healthy whereas items like pizza and chips were unhealthy as they were of poor nutritional value. Comparable views have been published previously wherein urban Chinese adolescents distinguished between healthy and unhealthy food based on its content and nutritional value.^(47)^ Our participants also discussed using different food groups like fruits, vegetables, milk, and milk products for attributing healthiness to a food item. Similar classification was also employed by Malaysian,^(32)^ American,^(48)^ as well as Swiss^(31)^ adolescents while perceiving healthiness of food products.

The adolescents perceived a particular food or beverage as being healthy or unhealthy depending on the immunity and health benefits provided by that item. Food items like fried snacks, burgers, and pizza were considered unhealthy because they contained high amounts of oil and fat which can cause multiple ailments including jaundice. Parallel findings were cited in a Zurich-based study whereby adolescents used fat and sugar content of the snacks to predict their healthiness.^(31)^ Our respondents also discussed that consumption of healthy food safeguarded the body against several diseases, a viewpoint also highlighted by Brazilian adolescents.^(49)^

Another frequently reported criterion for classifying nutritious or non-nutritious food was the type of ingredient used in its preparation. Packaged food like cakes, cookies, and chips were regarded as unhealthy because they were produced using harmful chemicals like preservatives, artificial colour, low-quality cooking oil, etc. Likewise, Chinese^(47)^ as well as Swiss^(50)^ adolescents also used this criterion while identifying healthy or unhealthy food products. A few of our participants also labelled food as healthy or unhealthy depending on the type of packaging material, for example, packaged foods were criticised for being packed in plastic wrapper. This finding was previously reported in a local study in which adolescents suggested replacement of plastic packaging with an eco-friendly substitute.^(51)^

In addition to the type of ingredient and packaging material, our study respondents also used location and time of food preparation for classifying healthy or unhealthy foods. They noted that anything prepared at home was healthy as it was prepared using local and fresh ingredients while foods available/prepared in the market could be stale as the time of preparation was not known; therefore, the food item may not be edible for consumption. In line with these findings, American adolescents also criticised the food available in fast food joints as well as reported that usually green vegetables are cooked at home by their parents and therefore labelled it healthy.^(25)^ Furthermore, in a recent cross-sectional study, urban Indian adolescents regarded the use of fresh ingredients in meal preparation as a facilitator to healthy eating.^(34)^

The study participants classified foods as healthy or unhealthy depending on their parents’ recommendations. They mentioned that their parents discouraged them from eating and purchasing unhealthy foods while encouraging them to consume healthy food like fruits and vegetables. In the same vein, Malaysian^(32)^ adolescents also reported that their parents controlled their food choices and recommended them to rectify their unhealthy food choices as well as promoted them to choose nutritious foods. Furthermore, in a Hong Kong based study, adolescents perceived their parents as an important socialising agent of healthy eating.^(52)^ Additionally, adolescents from urban India^(53)^ also echo similar views about parents being socialising agents for healthy eating. Interestingly, some studies suggest that parental influence on adolescents’ food habits begins to diminish during adolescence^(54,55)^ as it gets replaced by the more influential peer group.^(56,57)^ However, none of our respondents mentioned the motivating role of peers in classifying healthy and unhealthy food choices.

Important implications for nutrition education and public health policies can be derived from our results. School-based nutrition education programmes focusing on providing health and food literacy knowledge and skills to adolescents should be developed since culinary skills^(50,58)^ are regarded as indispensable life skills which will help adolescents to live healthy and independent lives.^(59–61)^ These strategies are undoubtedly warranted at a time when adolescent obesity and the associated chronic degenerative diseases are escalating in several low-middle-income economies like India. Consumer education for the masses including parents and teachers could be imparted through communication campaigns and social media enhancing both declarative and theoretical nutrition knowledge of individuals with the aim of improving their dietary behaviour including food selection.^(51,62)^

This is one of the first research investigations to provide first-hand information on Indian adolescents’ perceptions of healthiness and unhealthiness. The use of qualitative research techniques, i.e. in-depth face-to-face interviews, assisted in eliciting rich and novel data about adolescents’ food classification criteria. Nevertheless, these findings need to be treated cautiously. One of the limitations of this study is the use of convenience sampling strategy which could have affected the generalisability of our findings. Although the use of random sampling techniques might be regarded as ideal, qualitative researchers advocate the use of deliberate sampling techniques like convenience because the aim of this kind of research is not to generalise rather provide in-depth knowledge about the topic under investigation through a detailed inquiry.^(63,64)^ A further limitation is that the study findings may not be generalised to the general Indian adolescent population since our research inquiry was based mainly on a rural sample. This suggests the need to extend this inquiry to urban as well as different geographical settings owing to India's diverse dietary culture.

In conclusion, this formative research inquiry provided novel, rich, in-depth information about Indian adolescents’ perception of healthiness and unhealthiness of food. Adolescents used a number of criteria to classify foods as healthy or unhealthy which included nutrients and food groups, immunity, type of ingredient and packaging, location and time of food preparation, and parental influence. From this first-hand information, we can draw significant implications for future public health policies and nutrition education programmes. Skill-based nutrition education could be delivered in schools for instilling life skills like culinary skills in pupils which will encourage them to lead a heathy and independent adulthood.

## Supporting information

Kansal et al. supplementary material 1 Kansal et al. supplementary material

Kansal et al. supplementary material 2 Kansal et al. supplementary material

## Supplementary material

The supplementary material for this article can be found at https://doi.org/10.1017/jns.2023.101.
